# Alcohol-positive multiple trauma patients with and without blood transfusion: an outcome analysis

**DOI:** 10.1186/1752-2897-3-3

**Published:** 2009-03-06

**Authors:** Manuel F Struck, Thomas Schmidt, Ralph Stuttmann, Peter Hilbert

**Affiliations:** 1 Bergmannstrost Trauma Center, Department of Anaesthesiology, Intensive Care and Emergency Medicine, Merseburger Strasse 165, 06110 Halle (Saale), Germany; 2 Bergmannstrost Trauma Center, Department of Medical Psychology, Merseburger Strasse 165, 06110 Halle (Saale), Germany

## Abstract

**Background:**

Blood transfusion is a common therapy for multiple trauma patients, and is often performed soon after hospital admission. It is unclear whether the need for a blood transfusion in multiply injured patients presenting with a positive blood alcohol concentration (BAC) is associated with increased morbidity/mortality, since their risk behavior differs significantly from patients with a negative BAC. In this study, we evaluated the role of blood transfusion in the treatment of BAC-positive multiple trauma patients.

**Patients:**

In a three-year period, 164 patients at a single trauma center presented with a positive BAC, and 145 met the inclusion criteria for further evaluation and regression analysis. We compared patients who were transfused (n = 76) with those who were not transfused (n = 69).

**Results:**

In both groups, the most common causes of trauma were traffic accidents and falls. Most patients were admitted to the hospital from the scene of the accident (77.2%) and were male (89.0%). Transfused patients had a lower GCS (p ≤ .001) and her ISS (p ≤ .001), were more likely to have severe head injuries (p ≤ .001), tended to have higher BACs (p = .053), had lower hemoglobin levels and prothrombin times in the first 24 hours (p ≤ .001), had lower lactate levels, had higher rates of intubation (p ≤ .001) and ICU admission, and had longer ICU stays and artificial ventilation times (p ≤ .001). Mortality was significantly higher in transfused patients (n = 15 vs. n = 3, p ≤ .001). Non-survivors were more likely to have severe head injuries; be intubated and ventilated; be older; have higher ISS scores, lactate levels, and numbers of transfusions in the first 24 hours; and have lower GCS scores, hemoglobin measurements, and prothrombin levels. In a binary logistic regression model, only age (p = .009) and ISS (p = .004) independently predicted mortality.

**Conclusion:**

In our single-center study, the BAC of multiple trauma patients and the number of blood transfusions they received did not predict mortality in multiple trauma patients if used as independent predictors. Prospective studies with greater sample sizes should be performed to clarify the role of blood transfusions in the outcome of this sub-population.

## Background

Trauma patients with elevated blood alcohol concentrations (BAC) generally differ from other patients in terms of their risk behaviors, the likelihood of impaired consciousness, and their reaction times [1]. They are also more likely to have incomplete diagnoses in the emergency department and to have a higher rate of intensive care-related complications, such as pneumonia and longer artificial ventilation times [2,3]. Staff, misled by the impaired mental status of blood-alcohol positive patients, are often slower to instigate life-saving interventions [4]. The negative effects of a positive BAC may be amplified by a therapeutic intervention, such as blood transfusion, that is considered to be prognostic in a calculation model measuring outcome.

Although blood transfusion is a reliable predictor of increased risk of morbidity and mortality in multiple trauma patients [5-7], it can be life-saving, especially if there are effective and efficient re-warming procedures, aggressive correction of coagulopathy, timely and appropriate interventions to control blood loss, and modern blood banking procedures [8]. Even massive transfusion is justifiable in major trauma resuscitations [9-12].

Prospective randomized studies to evaluate the effects of blood transfusion in acute trauma care are very difficult to perform because of ethical and legal limitations. Transfusion protocols are often based on expert opinion and not on evidence [13], whereas studies focusing on the combination of a positive BAC and transfusion in multiple trauma patients are not published. In the present retrospective cohort study, the role of transfusion in morbidity and mortality of alcohol-positive patients with multiple traumas was evaluated. We hypothesized that a higher BAC and larger number of packed red blood cell transfusions would lead to higher morbidity and mortality.

## Methods

### Study population

In a three-year period (January 2004 to December 2006), data from 573 consecutive adult multiple trauma patients admitted to an urban trauma center (Bergmannstrost Trauma Center, Halle/Saale, Germany) were collected retrospectively; 206 patients underwent blood alcohol screening, and 164 tested positive. We evaluated the diagnostic, therapeutic, and outcome characteristics of these patients and compared patients who received blood transfusions with patients who did not.

Inclusion criteria were suspected high-impact trauma, hospital admission on trauma Day One, and a positive BAC. Exclusion criteria were major burns, absence of an emergency physician at the accident scene, or missing data. The study protocol was approved by the hospital's data security manager and the local ethics committee.

All patients were transported to the hospital by physician-staffed ground ambulance or helicopter rescue. Some patients came directly from the scene of the accident (primary admission), and others were transported from smaller hospitals where they had received their initial treatment (secondary admission). All patients had a whole-body multi-slice CT-scan (Toshiba Aquilion 32), in accordance with the center's trauma protocol. Retrospective analysis was performed using computerized medical records, focusing on socio-demographic data and trauma- and treatment-specific variables. Personal patient information was treated in accordance with the Declaration of Helsinki.

### Definitions of diagnostic and therapeutic variables

'Multiple trauma' was defined as either two or more injuries (life-threatening in combination) or one life-threatening injury [14]. An initial Glasgow coma scale (GCS) score was calculated at the scene of the accident by the emergency physician. A diagnosis of 'severe head injury' was made if there was a skull fracture or intracranial hemorrhage on head computed tomography (CT), as interpreted by a radiologist. The ISS was calculated after the initial resuscitation by a senior anesthesiologist (always the same person). Blood alcohol samples were taken in the emergency department. The lowest hemoglobin and prothrombin levels and the highest lactate levels in the 24 hours after admission were recorded. Airway management was classified into the following categories: tracheal intubation at the scene, in the emergency department, during transportation, or not performed. Admission and length-of-stay in the ICU, as well as the need for and duration of artificial ventilation, were noted. The number of packed red blood cell transfusions in the first 24 hours after admission and throughout hospitalization was calculated. For patients who died of their injuries, the time of survival after trauma was recorded.

### Statistics

Group comparisons were analyzed using the χ^2 ^test, Student's t-test, or the Mann-Whitney U-test. The alpha level of significance was set at 5%, two-tailed. To evaluate predictors for mortality, a binary logistic regression model was employed for the whole study population. Further exploratory analyses used Spearman rank correlations. All computations were made using SPSS 13.0.

## Results

### Study population

From the cohort of 164 patients with a positive BAC, 19 patients were excluded: nine did not meet the requirement for the definition of multiple trauma, four had major burns, four were admitted after trauma Day One, and two had incomplete data. In the emergency department, 45 of the 145 patients had a BAC of >0.5 g/l and 36 had a BAC of >1.0 g/l. Seventy-six patients received a blood transfusion (transfusion group [TG]), and 69 patients did not (no transfusion group [NTG]). Both groups were comparable with respect to age, and most patients in both groups were male (89% of the whole study population). The characteristics of the two groups and statistical comparisons are summarized in Table 1.

**Table 1:** Characteristics of patients with and without blood transfusion

*Measure*	*TG (n = 76)*	*NTG (n = 69)*	*P value*
Age, years, mean (SD)^a^	39.0 (18.2)	38.6 (17.0)	.885
Male, n [%]/female, n [%]^b^	63 [82.9]/13 [17.1]	66 [95.7]/3 [4.3]	.*014*
ISS, mean (SD)^c^	35.1 (17.5)	16.4 (14.0)	≤.*001*
GCS, mean (SD)^c^	9 .2 (4.9)	11.9 (4.6)	≤.*001*
BAC, g/l, mean (SD)^c^	.65 (.90)	.41 (.74)	.053
Severe head injury, n [%]^b^	44 [57.9]	20 [29.0]	≤.*001*
Mortality, n [%]^b^	15 [19.7]	3 [4.3]	.*005*
ICU admission, n [%]^b^	67 [88.2]	28 [40.6]	≤.*001*
ICU days, mean (SD) ^c^	16.0 (14.5)	3.5 (7.0)	≤.*001*
Artificial ventilation, n [%]^b^	61 [80.3]	18 [26.1]	≤.*001*
Artificial ventilation hours, mean (SD)^c^	273.8 (293.2)	47.0 (118.5)	≤.*001*
No intubation, n [%]^b^	17 [22.4]	44 [63.8]	≤.*001*
Hemoglobin, mmol/l, mean (SD)^a^	5.4 (1.3)	7.8 (1.2)	≤.*001*
Prothrombin, sec, mean (SD)^c^	72.7 (28.7)	95.7 (25.7)	≤.*001*
Lactat, mmol/l, mean (SD)^c^	3.1 (2.7)	1.9 (1.8)	≤.*001*
Trauma context, n [%]^b^			.603
Traffic accident	50 [65.8]	45 [65.3]	
Pedestrian	6 [7.9]	3 [4.3]	
Bicyclist	6 [7.9]	8 [11.6]	
Motorbiker	13 [17.1]	16 [23.2]	
Car driver	24 [31.6]	16 [23.2]	
Truck driver	1 [1.3]	2 [2.9]	
Fall from height	25 [32.9]	21 [30.4]	
Other	1 [1.3]	3 [4.3]	
Admission characteristics, n [%]^b^			
Primary from the scene/	51 [67.1]/	61 [88.4]/	.*002*
Secondary from other hospital	25 [32.9]	8 [11.6]	.125.
Helicopter total	46 [60.5]	33 [47.8]	.*012*
Helicopter secondary	19 [25.0]	7 [10.1]	

^a ^t test; ^b ^χ^2 ^test; ^c ^U test

TG = group of transfused patients; NTG = group of patients not transfused; SD = standard deviation; n = number of patients; ISS = Injury Severity Score; GCS = Glasgow coma scale; BAC = blood alcohol concentration in g/l, ICU = intensive care unit

### Injury cause and admission

The two groups did not differ in the causes of their trauma (Table 1): in both groups of patients, the major causes were traffic accidents and falls from a height. Four patients (n = 1 in TG vs. n = 3 in NTG) had other causes for their trauma: one patient was buried under a tumbling wall, two were injured in complex workplace accidents, and one was shot.

Most patients (77.2%) in both groups were primary admissions to the trauma center. Similar proportions of patients in both groups underwent helicopter rescue (table 1), but the TG patients were more likely to be secondary admissions (p = .002)

### Diagnosis and therapy

Transfused patients had lower GCS scores (p ≤ .001) and higher ISS (p ≤ .001) and were more likely to have a severe head injury (p ≤ .001). The BAC tended to be higher in TG patients (p = .053). Hemoglobin levels and prothrombin times in the first 24 hours were lower (p ≤ .001), and lactate levels were higher (p ≤ .001) in TG patients. Only 16 females were found in the study population; most of them received blood transfusions (81.3%).

Higher BACs correlated with lower GCS scores, but not with other clinical variables (correlations between BAC and age, laboratory results, ISS, transfusion amount, duration of intensive care, artificial ventilation, and days to decease in non-survivors were low and not significant).

The overall rate of tracheal intubation was 57.9%. Transfused patients had a higher rate of tracheal intubation, especially at the accident scene and in the emergency department. The number of transport-related intubations was similar in both groups. Furthermore, transfusion was associated with a higher admission rate to the ICU, longer ICU stays (p ≤ .001), and longer artificial ventilation times (p ≤ .001).

The average number of transfusions was four in the first 24 hours and 12 throughout hospitalization (Table 2). Twelve patients received massive transfusions (>10 units) in the first 24 hours. Only the laboratory parameters were significantly different between massively transfused patients and patients receiving lesser amounts of blood (lower hemoglobin and prothrombin time, higher lactate; p ≤ .001). BAC, age, ICU admission length, artificial ventilation times, GCS, and ISS were not related to transfusion amount.

**Table 2:** Amounts of transfused erythrocytes concentrates

*Transfusion (n = 76)*	*Minimum*	*Maximum*	*Mean*	*Standard deviation*
Within 24 h	0	22	4.03	4.91
During hospitalization	2	64	12.72	11.08

### Outcome

Eighteen patients did not survive. Mortality was significantly higher in transfused patients (n = 15 in TG vs. n = 3 in NTG, p ≤ .001). The trauma contexts of deceased patients were traffic accident (n = 8), fall from height (n = 9), and shotgun injury (n = 1). Two patients died after discharge from the ICU. Non-survivors were more likely to have had a severe head injury and to have undergone tracheal intubation and artificial ventilation.

Significant differences between non-survivors and survivors were: ISS, age, intubation rate, lactate level, number of transfusions in the first 24 hours and total number of transfusions, GCS scores, and hemoglobin and prothrombin levels. BAC, ICU admission, length of ICU stay, and hours of artificial ventilation were equal in both groups. There were no differences in gender, admission type, or likelihood of severe head injury.

A binary logistic regression model was used to examine predictor variables for mortality (Table 3). The potential predictors assessed were age, GCS score, ISS, laboratory results, intubation, artificial ventilation, head injury, and transfusion. Since BAC must be considered as a confounder, we included it as an independent variable in the regression model. With a given fit of the regression model, only two parameters predicted mortality, age (p = .006) and ISS (p = .003), and these correctly predicted mortality in 66.7% of cases and survival in 98.9% of cases. If only TG (where most patients died) was used for predictive analysis, age (p = .023) and ISS (p = .006) remained the only significant predictors; they predicted mortality in 61.5% of cases and survival in 98.2% of cases.

**Table 3:** Predictors for mortality in a binary logistic regression model

*Measures*	*Odds ratio*	*95% Confidence intervals*	*P value*
Age	1.08	[1.02; 1.15]	.*006*
ISS	1.12	[1.04; 1.21]	.*003*
Hemoglobin	.98	[.44; 2.2]	.959
Prothrombin	1.02	[.98; 1.07]	.305
Lactate	1.53	[.98; 2.38]	.062
Artificial ventilation	4.13	[.02; 1051.39]	.616
Transfusion	.38	[.02; 7.75]	.529
Intubation	.201	[.01; 4.04]	.295
GCS	.86	[.67; 1.1]	.226
Severe head injury	2.03	[.19; 22.14]	.56
BAC	.61	[.18; 2.04]	.423

ISS = Injury Severity Score; GCS = Glascow Coma Scale; BAC = blood alcohol concentration in g/l

## Discussion

Mortality was higher in transfused patients, but whether or not a patient was transfused was not a predictor of outcome in patients with a positive BAC. The higher mortality rate in these patients probably reflected the underlying severity of their injuries, with the need for transfusion being a marker of this.

In our study, the BAC level did not predict mortality. One previous study found that a higher BAC is a risk factor for poor outcome in brain injury patients [15]. Recent studies suggest that BAC neither influences initial GCS scores nor weakens the prognostic power of commonly used predictive laboratory measures, such as a base deficit and lactate, despite investigations showing higher values in alcohol-positive patients [16-20].

In a retrospective study of adult multiple trauma patients with an ISS > 12, a positive BAC combined with the presence of an illegal substance was associated with a higher rate of pneumonia and longer ventilation time, whereas alcohol alone did not exhibit those associations [21]. There is no proven link between BAC and particular injury patterns, but there may be a tendency for a higher rate of head injury [22,23]. In our study population, head injury was not associated with BAC.

In prospective studies on alcohol-impaired patients involved in traffic accidents, Fabbri found the following independent risk factors for severe injury: male sex, older age, admission at night or over a weekend, and a BAC >0.5 g/l [2,3,24]. These patients were very likely to be re-admitted to the emergency department with injuries sustained in a later traffic accident or violent incident [24]. Another study revealed that BAC-positive patients with severe head injuries had delays of more than two hours in receiving intracranial pressure devices, delays which could be detrimental [4]. Furthermore, the rate of incomplete diagnoses seems to be significantly higher in BAC-positive patients [2]. Reasons for underestimating the severity of their condition may include their altered mental status or the different diagnostic approaches used with those patients. In our study, all patients underwent uniform, rapid, whole-body CT, which has previously been demonstrated to be a reliable, accurate, and timesaving assessment method [25].

One problem in retrospective studies involving BAC or illegal drugs in trauma is that many centers do not routinely screen for these. Only half of trauma patients are tested for alcohol, and even fewer are tested for other abused substances [26]. Increasing costs and legal considerations may be restricting the use of these tests [26]. In our study, only 36% of trauma patients had blood alcohol screening. We did not include a control-group with a negative BAC because of the low sample size.

There was a higher percentage of secondary admissions in the transfused group. This may reflect a lower quality of care from less specialized centers or delays in receiving definitive treatment. Laboratory measures are helpful physiological instruments for predicting trauma severity in clinical practice. Hemoglobin as a marker of anemia, lactate as a correlate of anaerobic metabolism, and prothrombin time as a marker of coagulation status are commonly used in multiple trauma outcome studies [27,28]. In our study population, none of the laboratory tests predicted mortality. Unlike hemoglobin and prothrombin testing in both of our patient groups, lactate appeared to have been assessed in only 50% of NTG cases and was therefore not useful in predictive analyses.

In larger, North American study populations, blood transfusion in the first 24 hours after trauma was an independent predictor of mortality, ICU admission, length of ICU stay, and systemic inflammatory response syndrome (SIRS) [29,5]. We likewise found that receiving a blood transfusion increased the likelihood of an ICU admission and the risk of longer ICU stays and durations of artificial ventilation. However, even massive transfusion did not appear to be a prognostic factor for mortality in our patients. Transfused patients were significantly more likely to undergo tracheal intubation, a variable that correlates with increasing injury severity patterns.

Evaluating the role of blood transfusions in severe trauma is of great public concern because the conclusions may affect patient safety and public spending on blood products [30]. Restrictive transfusion strategies can reduce transfusion related-complications (such as SIRS or transfusion-related lung injury (TRALI)) and reduce costs, but there is currently no real alternative to blood transfusion in the treatment of severe traumatic hemorrhage [7,29]. Multidisciplinary guidelines therefore include transfusion as an important part of trauma resuscitation [28,31].

### Limitations

Our study has several important limitations. Its retrospective character could have influenced the availability of the data collected. The decision to transfuse packed cells was at the discretion of the attending intensivist and not based on transfusion protocols or scoring systems [13,32,33]. Although regression analysis minimizes confounding in cohort studies, only prospectively performed randomization of patients to standard protocols can reduce the effects of subjective assessment and treatment by physicians [34]. Additionally, our patients were not a homogeneous group, but rather presented with a variety of trauma etiologies. Also, our data reflect the experience of a single trauma center and are drawn from a relatively small sample size.

There was a large number of patients transferred from other facilities in whom BAC-determination was performed relatively late in the course of their management, and this may have led to unreliable laboratory results (such as false negatives, with BAC having declined to undetectable levels) [35]. Finally, the low number of other potential predictive variables (e.g., patient hemodynamics, infusion of vasoactive agents, or the presence of organ failure) limits the general power of the study.

Nevertheless, the present study provides data in an under-researched field. Our findings suggest that morbidity is higher in transfused BAC-positive trauma patients and that transfusion use in BAC-positive patients correlates strongly with poorer laboratory measures and higher expenditure resulting from an increased need for intensive care. We found that ISS and age were robust predictors of overall mortality.

## Conclusion

We conclude that the level of a multiple trauma patient's BAC and the amount of packed red blood cells transfused did not predict mortality. However, more transfusions were correlated with higher injury severity and comorbidity. Prospective studies with higher sample sizes should be performed to clarify the role of blood transfusion in the outcome of this sub-population.

## Competing interests

The authors declare that they have no competing interests.

## Authors' contributions

MFS designed the study, collected data, reviewed the literature, and drafted the manuscript. TS performed the statistical analysis and helped to draft the manuscript. RS participated in the study design. PH collected data, participated in the study design, and helped to draft the manuscript. All authors read and approved the final manuscript.
